# Health-related quality of life and nursing-sensitive outcomes in mechanically ventilated patients in an Intensive Care Unit: a study protocol

**DOI:** 10.1186/s12912-016-0127-9

**Published:** 2016-02-05

**Authors:** Alba Riera, Elisabet Gallart, Araceli Vicálvaro, Montserrat Lolo, Anabel Solsona, Anna Mont, Jordi Gómez, David Téllez, Carmen Fuentelsaz-Gallego

**Affiliations:** Intensive Care Unit, Hospital Universitari Vall d’Hebron, Universitat Autònoma de Barcelona, Barcelona, Spain; Intensive Care Unit, Hospital Universitari Vall d’Hebron, Barcelona, Spain; Hospital Universitari Vall d’Hebron, Universitat Autònoma de Barcelona, Barcelona, Spain

**Keywords:** Critical care, Mechanical ventilation, Quality of life, Stressors, Nursing sensitive outcomes, Nursing

## Abstract

**Background:**

Mechanical ventilation (MV) is one of the most utilised techniques in the intensive care unit (ICU), but it can cause sequelae that can negatively influence the patient’s health-related quality of life (HRQL). Nursing-sensitive outcomes (NSOs) can also influence the HRQL. Assessing the HRQL of mechanically ventilated patients admitted to an ICU and its relation to nurse-sensitive outcomes will give healthcare professionals with valuable information to improve patient care.

**Methods:**

Prospective longitudinal cohort study in which all patients admitted to the ICU at *Hospital Universitari Vall d’Hebron* who undergo MV for more than 48 h will be included. The study will last 12 consecutive months. HRQL will be assessed by the completion of the SF-36 and the Saint Georges Respiratory Questionnaire. Pre-admission HRQL assessment will be performed by the main caregiver, and after ICU discharge, the assessment will be performed by the patient him/herself. The same questionnaires will also be completed one year after ICU discharge. Other variables (sociodemographic and those related to reason for ICU admission, ICU length of stay, MV, ICU stressors and NSO) will be included in a multiple regression model to assess their relation to the patient’s HRQL.

**Discussion:**

This study will show the relationship between the HRQL perceived by patients and their main caregiver, what the HRQL is one year after discharge from ICU, and what the impact of MV, NSO and ICU stressors and other clinical outcomes on the patient’s HRQL is. Determining mechanically ventilated patients’ HRQL and its relation to NSO and ICU stressors as well as other clinical variables will enable early nursing interventions to try to minimise possible sequelae and improve the patient’s welfare.

**Trial registration:**

*ClinicalTrials.gov ID:*NCT02636660*Registration Date:* 17th December 2015.

## Background

Mechanical ventilation (MV) is one the most utilised therapeutic techniques within the intensive care unit (ICU). It is commonly used to support patients diagnosed with severe respiratory failure [1], but several complications have been documented in the literature [2] that can appear during both the acute phase of treatment and in later phases, becoming a chronic problem in some cases. Most of these complications are related to the length of treatment [3–5], and they could affect patients’ health-related quality of life (HRQL).

HRQL conceptualisation is vital for the analysis and evaluation of health-related outcomes. Knowing the patient’s health perception, his wishes and motivating factors when making decisions related to his own health as well as applying procedures to evaluate healthcare providers, is essential to the definition of HRQL.

Diagnosis and treatment of illness exclusively at a biomedical level in addition to technologically advanced procedures represent a qualitative improvement in patient survival in recent decades, but they have reduced a more holistic approach to health care because they only fight against illness without promoting patient welfare. From a more holistic perspective, and in an attempt to quantify the contribution of nursing to health-related outcomes, the term “nursing-sensitive outcome” (NSO) was created. According to the National Quality Forum, those indicators that are sensitive to certain nursing interventions are measurements of the process’ structure (the process itself and its outcomes) and are affected or influenced by the intervention of nurses, although the responsibility is shared with other professionals. The forum agreed on several NSO standards such as pressure ulcer care, infection control, falls prevention, expertise of the nursing team, patient satisfaction and HRQL [6].

Outcome evaluation and knowledge of HRQL in mechanically ventilated critical care patients is vital to understand how this therapeutic technique affects patients and therefore designing care strategies for both acute and later phases of treatment. The goal is to minimize unwanted effects and improve cost-effectiveness, always considering the patient’s perspective during the decision-making process.

The analysis of factors that determine the patient’s perception on HRQL during the different phases of illness as well as the adaptation process of unwanted results will give us in-depth knowledge of the mechanisms that affect their HRQL in a negative way and therefore plan interventions to promote the optimal patient welfare [7].

There is a great deal of published literature on mechanically ventilated patient’s HRQL. All authors agree that the quality of life perceived by these patients is worse than that perceived by the reference population [4, 8–11], although determinants of HRQL are not well defined. Additionally, differences among critical care units (surgical, cardiac, general) and the variety of tools utilised to measure HRQL make it hard to compare results [11].

Highly specialised ICUs constitute a very stressful environment for the patient, which has been related to the onset of delirium or an acute confusional state in different phases of the disease, but other mid and long-term repercussions such as memory loss, delusion and other neurophysiological disturbances that have an effect on HRQL perception have been described [12–14].

Some authors have studied factors that have a stressful effect on mechanically ventilated patients [15–18] and have found that one of the worst remembered and most stressful experiences, aside from thirst and pain, is endotracheal tube discomfort [19]. There are several tools to assess stressors within ICU, and all of them allow us to assess the degree of stress or nuisance experienced by the patient for each factor. One of the most well-known is the Intensive Care Unit Environmental Stressor Scale (ICUESS), which allows us to perform a global evaluation of the ICU but does not take into account problems secondary to tracheal intubation or MV. Another tool recently described in a HRQL study on mechanically ventilated patients is the ICU Stressful Experience Questionnaire (ICU-SEQ), which evaluates the general aspects of an ICU setting but also emphasizes those problems that originated as a result of orotracheal intubation and MV [12, 14, 15].

Knowing what HRQL is, its stressors and which NSO have an influence on it should allow us to design and implement strategies and nursing care plans to minimise negative effects and address chronic problems, thereby improving the patient’s welfare, security and quality of life.

## Methods/design

### Aims

#### Main objective

To assess health-related quality of life of mechanically ventilated patients admitted in an ICU and its relation to nursing-sensitive outcomes.

#### Secondary objectives

1. To determine whether there are any differences in HRQL in mechanically ventilated patients prior to admission into ICU and one year after discharge from the ICU.
2. To identify whether there is any relationship between the duration (in days) of mechanical ventilation and HRQL one year after discharge from the ICU.
3. To identify whether there is any relationship between NSO and HRQL of mechanically ventilated patients one year after discharge from the ICU.
4. To identify differences between the patient’s own assessment of HRQL and the main caregiver’s perception.
5. To identify stressors that mechanically ventilated patients admitted in ICU identify when discharged and which ones last for at least one year after they have left the ICU.
6. To identify whether there is a relationship between the amount and type of stressors perceived by mechanically ventilated patients during their admission and their HRQL one year after discharge from the ICU.

### Design

This is a prospective longitudinal cohort study.

### Setting

The study will be carried out at the Intensive Care Unit of the *Hospital Universitari Vall d’Hebron*, located in Barcelona, Spain, which is a high-complexity hospital and a referral centre for many disease processes at a national level.

### Subjects of study

All mechanically ventilated patients who fulfil the selection criteria outlined below were enrolled in the study:

#### Inclusion criteria

- Over 18 years old.
- Mechanical ventilation for more than 48 h.
- Voluntary participation (signed informed consent).

#### Exclusion criteria

- Patients admitted from other ICUs that had been mechanically ventilated for more than 48 h at the time of admission to our centre.
- Patients with domiciliary respiratory support.
- Mentally or psychologically impaired patients (unable to comprehend the questionnaire).
- Patients who do not speak Spanish fluently.
- Patient without a permanent address (unable to do a proper follow-up).

#### Sample size

According to data previously collected in our department, a total of 360 patients should meet the inclusion criteria during the study timeframe. Assuming possible losses and a high mortality rate within critical care, the final cohort is expected to be 200 patients.

#### Sampling technique

A non-probabilistic convenience sampling will be used, including patients as they are admitted to the ICU.

### Variables

The following variables will be collected: anthropometric and sociodemographic data and data related to ICU admission, mechanical ventilation, NSO, HRQL and ICU stressors. Variable definitions and the data collection schedules are summarized in Table 1

**Table 1:** Variables and Measurement Schedule

Category	Variables	Measurement Schedule
Anthropometric	Age, Gender	Admission
	Weight, Height	Admission and 12 months after ICU discharge
Sociodemographic	Academic level	Admission and 12 months after ICU discharge
	Employment, economic income	
	Main caregiver, family/social support system	
ICU length of stay	Main medical diagnosis, reason for ICU admission, APACHE II, Age corrected Charlson Index	Admission
	ARDS, MODS, Surgical interventions, blood transfusion, renal replacement therapy, Ramsay Score for >3 hours.	Daily
	Complications, GCS, Barthel Index, length of ICU stay (days) and hospital stay	ICU discharge and 12 months after
		ICU discharge and hospital discharge
Mechanical Ventilation	Reason for intubation and MV	Admission
	Modes of MV utilised (hours and days)	Daily
	Respiratory complications	
	Days with tracheostomy tube in place	ICU Discharge
	Days on MV	
NSO	Total hours in pain (Assessed using a VAS), PU, Falls, VAP, CRB, UTI	Daily
	Allocated nurse’s expertise	
HRQL	SF-36, QRSG	ICU discharge and 12 months after
	SF-36, QRSG (assessed by main caregiver)	ICU discharge
ICU Stressors	ICU-SEQ	ICU discharge and 12 months after

APACHE II: Acute Physiologic A Chronic Health Evaluation II, ARDS: Acute Distress Respiratory Syndrome, MODS: Multiorgan Dysfunction Syndrome, GCS: Glasgow Coma Score, ETT: Endotracheal Tube, MV: Mechanical, VAS. Visual Analog Scale, PU: Pressure Ulcers, VAP: Ventilator Associated Pneumonia, CRB: Catheter Related Bacteriemia, UTI: Urinary Tract Infection, SF-36: Medical Outcome Study-Short Form 36, QRSG: Saint Georges Respiratory Questionnaire, ICU-SEQ: ICU Stressful Experience Questionnaire

.

### Assessment tools

To assess HRQL, the Short-Form-36 Health Survey (SF-36) will be used. For a more specific assessment on respiratory issues, the Saint Georges Respiratory Questionnaire (SGRQ) will be used. Both questionnaires have been validated in Spanish.

To evaluate ICU stressors, the ICU Stressful Experience Questionnaire (ICU-SEQ) will be used. This tool was created by Rotondi and modified by Samuelson. In addition to evaluating general ICU experiences, it also evaluates experiences related to the endotracheal tube.

To translate and adapt the ICU-SEQ into Spanish, the following process will be used:

1. Translation and back translation into Spanish by two bilingual translators.
2. Transcultural adaptation via a pilot study to assess proper comprehension of the different items.

Details of the assessment tools and data collection schedule are summarized in Table 2

**Table 2:** Measurement tools

Tools	Assessment Area	Dimensions	Items	Score
SF-36	Health-Related Quality of Life (general)	Physical sphere, social sphere, Physical role, Emotional role, mental health, vitality, pain, general health (8)	36	Every item of each dimension (8 in total) will be codified, aggregated and turned into a scale, with values that range from 0 till 100 (rating each health-related dimension from worse to best)
QRSG	Health-Related Quality of Life (respiratory)	Symptoms, activity, impact (3)	50	Each item has a specific weight assigned on the global score. Scores can range from 0 till 100 (rating quality of life from minimal to maximal alterations).
ICU-SEQ	ICU Stressors	No dimensions as such, but 2 very differentiated areas: ICU stressors in general and airway related stressors.	31	It’ll be assess if the patient remembers anything, and if so, assessment of stress’s perception will be performed (range of values: none, very little, Some, Quite a bit, A lot)

SF-36: Medical Outcome Study-Short Form 36, QRSG: Saint Georges Respiratory Questionnaire, ICU-SEQ: ICU Stressful Experience Questionnaire

.

### Data collection

Data will be collected over 12 consecutive months. Patient selection for the study cohort will be performed by the research team by evaluating all patients who may need or had needed MV. Follow up documentation will be provided for all mechanically ventilated patients. When a patient has been mechanically ventilated for over 48 h, informed consent will be obtained from the next of kin. From that moment, all patient data from their medical records will be gathered until MV is discontinued.

One week after the patient has been discharged from the ICU, informed consent will be obtained from the patient. Approaching the patient earlier is considered to be inappropriate by the research team. If the patient agrees to participate in the study, the SF-36, SGRQ and ICU-SEQ questionnaires will be provided for the patient to complete. The same questionnaires will be given to the main caregiver at that time. One year after ICU discharge, the same questionnaires will be completed again to assess the persistency of stressors’ sequelae.

### Data analysis

A descriptive analysis will be carried out for all variables. Mean and standard deviation will be utilised for quantitative variables that follow a normal distribution. For the rest of the variables, the median, maximum and minimum values will be used. Qualitative variables will be described utilising absolute frequencies and percentage values for each category.

Statistical analysis of HRQL results will be performed on the questionnaire’s different dimensions as well as on the questionnaire as a whole. To compare the results obtained at the time of ICU discharge with those obtained one year after discharge, Student’s t-test or Wilcoxon test will be applied to the paired data depending on the variable distribution. This analysis will also depend on the length of MV.

A multiple regression model will be elaborated utilising HRQL as the result variable. Relevant clinical situations (number of transfusions and surgical interventions, pressure ulcers, nosocomial infections…) will be considered as independent variables. All analysis will be performed with a 95 % confidence interval.

### Limitations and bias

The main limitation of this study is that the quality of life one year after ICU discharge could be affected by non-identifiable events that have no relation to those studied. The main bias is the patient’s memory because the patients will be asked about events that may be difficult to recall.

### Ethical considerations

Ethics approval was given by the Vall d’Hebron University Hospital Clinical Research Ethics Committee and Research Project Commission Report (Barcelona, Spain): Project number PR(AG)136/2011. Informed consent will be obtained from patients and main caregivers, always making sure participants’ confidentiality and anonymity is preserved through the process.

## Discussion

Advances in intensive care medicine have allowed us to decrease mortality rates amongst patients admitted to ICU, although for some patients who have overcome a critical illness, their time in the ICU may have led them to have a life with major limitations and obstacles.

It is necessary to identify strategies that allow us to determine HRQL of patients prior to ICU admission because healthcare professionals are not able to determine it. And eventhough a few people filled in a questionnaire on HRQL prior to their ICU admission, most of the time they are unable to fill one out because of their condition at the time of admission.

This study proposes an indirect approach to patient’s HRQL perception by measuring HRQL perceived by the main caregiver. If there is a positive concordance between main caregiver and patient’s HRQL perception, this indirect measurement would provide the patient’s vision of his own healthcare as well as his thoughts on his health-disease process. Some studies state that critically ill patient’s preferences regarding advanced life support therapies are conditioned by their cost and handicaps that may result from ICU admission [20].

Being able to identify which factors negatively influence a patient’s HRQL, whether they are certain invasive therapies (MV or renal replacement therapies) or the stressful ICU environment, should allow us to establish proactive strategies that will prevent or diminish the occurrence of physical or psychological sequelae. It would be very beneficial to implement measures to prevent the onset of anxiety, post-traumatic stress disorder or depression, as well as establishing early rehabilitation programmes, instead of waiting for these disorders to appear. It is very important to know each therapeutic action’s specific impact in order to implement specific strategies during both the acute and later phases of the process, so adverse events resulting from therapeutic interventions as well as ICU-generated stress could be minimised, always taking into account the patient’s own perception during the decision-making process. This project will allow us to determine the perceived HRQL one year after discharge from ICU and compare it with that measured earlier on in the process, as well as analyse the negative experiences they had while in the ICU as a result of MV and their impact over time. It will also allow us to assess the effect of NSO and ICU stressors on health-related outcomes and how they affect HRQL. This study could highlight the importance of early nursing interventions, in both acute and later phases of the process, in order to minimise some of the sequelae and improve patient welfare.

It could also be useful to understand the concordance between the HRQL perceived by patients and main caregivers to understand the patient’s own motivations regarding his health process.

## Abbreviations

HRQL: health related quality of life
ICU: Intensive Care Unit
ICU-SEQ: Intensive Care Unit Stressful Experience Questionnaire
MV: mechanical ventilation
NSO: nursing sensitive outcomes
SGRQ: Saint Georges Respiratory Questionnaire
